# The role of *Elateriospermum tapos* yoghurt in mitigating high-fat dietary cause of maternal obesity—an experimental study

**DOI:** 10.3389/fendo.2023.1131830

**Published:** 2023-06-21

**Authors:** Ruth Naomi, Rusydatul Nabila Mahmad Rusli, Fezah Othman, Santhra Segaran Balan, Azrina Zainal Abidin, Hashim Embong, Soo Huat Teoh, Azmiza Syawani Jasni, Siti Hadizah Jumidil, Hasnah Bahari, Muhammad Dain Yazid

**Affiliations:** ^1^ Department of Human Anatomy, Faculty of Medicine and Health Sciences, Universiti Putra Malaysia, Serdang, Malaysia; ^2^ Department of Biomedical Sciences, Faculty of Medicine and Health Sciences, Universiti Putra Malaysia, Serdang, Malaysia; ^3^ Department of Emergency Medicine, Faculty of Medicine, Universiti Kebangsaan Malaysia, Kuala Lumpur, Malaysia; ^4^ Advanced Medical and Dental Institute, Universiti Sains Malaysia, Penang, Malaysia; ^5^ Department of Medical Microbiology and Parasitology, Faculty of Medicine and Health Science, Universiti Putra Malaysia (UPM) , Serdang, Malaysia; ^6^ Centre for Tissue Engineering and Regenerative Medicine, Faculty of Medicine, Universiti Kebangsaan, Kuala Lumpur, Malaysia

**Keywords:** probiotic, maternal programming, obesity, natural food product, inflammation

## Abstract

Maternal obesity is the key predictor for childhood obesity and neurodevelopmental delay in the offspring. Medicinal plants are considered to be the safe and best option, and at the same time, probiotic consumption during pregnancy provides beneficial effects for both the mother and the child. Current research has shown that *Elateriospermum tapos* (*E. tapos*) yoghurt is safe to consume and consists of many bioactive compounds that can exert an anti-obesity effect. Thus, this study has been designed to study the role of *E. tapos* yoghurt in mitigating maternal obesity. In this study, a total of 48 female Sprague Dawley (SD) rats were assigned to six groups, with eight rats per group, and obesity was induced over 16 weeks with a high-fat diet (HFD) pellet. On the 17th week, the rats were allowed to mate and pregnancy was confirmed through vaginal smear. The obese induced group was further divided into negative and positive control groups, followed by *E. tapos* yoghurt treatment groups with three different concentrations (5, 50, and 500 mg/kg). The changes in body weight, calorie intake, lipid profile, liver profile, renal profile, and histopathological analysis were measured on postnatal day (PND) 21. The results show that the group with the highest concentration of *E. tapos* yoghurt (HYT500) supplementation shows gradual reduction in body weight and calorie intake on PND 21 and modulates the lipid level, liver, and renal enzymes to a normal level similar to the normal group. In histological analysis, HYT500 reverses the damage caused by HFD in liver and colon, and reverses the adipocytes’ hypertrophy in retroperitoneal white adipose tissue and visceral fat. In conclusion, supplementation of *E. tapos* yoghurt during the gestational period up to weaning is effective in the gradual weight loss of maternal obese dams from the 500-mg/kg-supplemented group in this study.

## 1 Introduction

Maternal obesity is currently alarming and often ignored due to lack of knowledge on its long-term effects for both mothers and the fetus. A pre-pregnancy body mass index of more than ≥30 kg/m^2^ is termed maternal obesity (1). Maternal obesity is the key predictor for childhood obesity and often associated with neurocognitive development of the fetus. This could be due to the shared genetic polymorphism between the obese mother and the child or changes of epigenome in the child (2). The prevalence rate for obesity has tripled over the past few decades and more than 41 million children aged less than 5 years have been classified as obese globally (3). Studies show that the prevalence rate is increasing mostly in low- and middle-income countries while Asia leads the list with the highest documented cases for child obesity. The rising trends show that at least 85% of the adults will be obese by the year 2030. The cost of treating obesity-related comorbidity is distressing and requires urgent medical attention (4). Although countless measures have been taken to manage obesity, it is impossible to curb obesity completely since the root cause lies within the maternal subjects. Current available treatments such as metformin (5) are known to cause adverse gastrointestinal effects such as nausea, vomiting, and diarrhea (6) while moderate to vigorous exercise during pregnancy could induce hypoxia, hyperthermia, or even abnormal heart rate in the growing fetus (7).

Therefore, medicinal plants have been documented as the best option to treat maternal obesity through a pharmacological approach. Among these, *Elateriospermum tapos* (*E. tapos*), a tropical plant, has been proven to exhibit an anti-obesity effect due to its natural antioxidant property that is able to inhibit lipid peroxidation (8). *E. tapos*, also locally known as buah perah, commonly found in the deep forest of Southeast Asia, contains a wide range of bioactive compounds that has the potential to prevent accumulations of fats. Some of the identified bioactive compounds include tannins, alkaloids, linolenic acids, polyunsaturated fats, saponins, and sterols (9). Concomitantly, we found that consumption of probiotic during pregnancy improves the metabolism in the pregnant mother and reduces the risk of maternal obesity complications (10). Thus, we speculate that the consumption of medicinal plant-integrated yoghurt will give out more beneficial effects for both obese mothers and the child by mitigating maternal obesity and its complication. A recent study proves that it is safe to consume up to 2000 mg/kg of *E. tapos* yoghurt daily (9). Hence, this study has been designed to investigate the effect of *E. tapos* yoghurt in the mitigation of maternal obesity through various parameters.

## 2 Materials and methods

### 2.1 Collection and confirmation of *E. tapos* seeds

The fresh *E. tapos* seeds were obtained from the Forest Research Institute of Malaysia (FRIM), Pahang and were sent to the Herbarium Biodiversity Unit at the Institute of Bioscience, Universiti Putra Malaysia for purity check. The approved voucher code is UPM SK 3154/17.

### 2.2 Ethanol extraction of *E. tapos* seeds

About 500 g of *E. tapos* seeds was cleaned with running tap water and were soaked in 2 L of 95% ethanol for 7 days in room temperature. On the 7th day, it was filtered using filter paper (Whatman No. 1) to obtain the crude extract and was filtered again using a rotary evaporator (11). The precipitate collected is then mixed with maltodextrin powder in the ratio of 1:1 before proceeding with an overnight oven-drying process (12).

### 2.3 Formulation of *E. tapos* yoghurt

The yoghurt was prepared according to the guideline described elsewhere (13). Full cream milk (100 ml; Dutch Lady Purefarm UHT) was boiled at 75°C followed by cooling down at room temperature for 15–20 min. The starter culture consisting of S*treptococcus thermophilus APC151* and *Lactobacillus delbrueckii*subsp. *Bulgaricus ATCC 11842* was added to the milk and was incubated in a Pensonic PYM-700 yoghurt maker for 7–8 h. The final produced yoghurt was refrigerated overnight at 4°C and *E. tapos* powder was added to the yoghurt in the ratio of 2 g/100 ml (13).

### 2.4 Active compounds screening for *E. tapos* yoghurt using UHPLC-QTOF-MS

The *E. tapos* yoghurt fraction was evaluated using UHPLC. UHPLC was performed on an ACQUITY UPLC I-Class system from Waters, consisting of a binary pump, a vacuum degasser, an auto sampler, and a column oven. Phenolic compounds were chromatographically separated using a column ACQUITY UPLC HSS T3 (100 mm × 2.1 mm × 1.8 μm) also from Waters, maintained at 40°C. A linear binary gradient of water (0.1% formic acid) and acetonitrile (mobile phase B) was used as mobile phase A and B, respectively. The mobile phase composition was changed during the run as follows: 0 min, 1% B; 0.5 min, 1% B; 16.00 min, 35% B; 18.00 min, 100% B; and 20.00 min, 1% B. The flow rate was set to 0.6 ml/min and the injection volume was 1 μl (14).

### 2.5 TWIMS-QTOFMS analysis for *E. tapos* yoghurt

The UHPLC system was coupled to a Vion IMS QTOF hybrid mass spectrometer from Waters, equipped with a Lock Spray ion source. The ion source was operated in negative electrospray ionization (ESI) mode under the following specific conditions: capillary voltage, 1.50 kV; reference capillary voltage, 3.00 kV; source temperature, 120°C; desolvation gas temperature, 550°C; desolvation gas flow, 800 L/h, and cone gas flow, 50 L/h. Nitrogen (>99.5%) was employed as desolvation and cone gas. Data were acquired in high-definition MS^E^ (HDMS^E^) mode in the range m/z 50–1500 at 0.1 s/scan. Thus, two independent scans with different collision energies (CE) were alternatively acquired during the run: a low-energy (LE) scan at a fixed CE of 4 eV, and a high- energy (HE) scan where the CE was ramped from 10 to 40 eV. Argon (99.999%) was used as collision-induced dissociation (CID) gas (14).

### 2.6 High-fat diet

The high-fat diet was prepared with 17% protein, 40% fat, and 43% carbohydrate, containing 414 kcal/100 g. All ingredients were then blended with 6% corn oil (Vecorn, Yee Lee Corporation Berhad, Kuala Lumpur, Malaysia), 6% ghee [Crispo, Crispo-Tato (M) Sdn Bhd, Kuala Lumpur, Malaysia], 20% milk powder (Dutch lady, Dutch Lady Milk Industries Berhad, Selangor, Malaysia), and 68% standard chow pellet [Gold Coin Feedmills (M) Sdn Bhd, Selangor, Malaysia]. Standard chow pellet contains 306.2 kcal/100 g with 21% protein, 3% fat, and 48.8% carbohydrate (15, 16).

### 2.7 Experimental animals

All animal studies were performed upon obtaining approval from the Institutional Animal Care and Use Committee (IACUC), Universiti Putra Malaysia. The animal ethics committee granted approval for this study under the code UPM/IACUC/AUP-R025/2022. In this study, 48 female Sprague Dawley (SD) rats weighing between 200 and 250 g were used. All rats were acclimatized for 1 week at 22 ± 3°C at 12/12 h light/dark. All rats were supplemented with standard rat chow for the first week with free access to water.

### 2.8 Obesity induction

A total of 40 rats were supplemented with HFD for 16 weeks and 8 rats were fed with standard chow pellet *ad libitum*. Body weight and 24-h food intake (kJ) were recorded weekly for all rats. After 16 weeks, obesity was confirmed by measuring the difference of mean body weight between HFD- and standard chow pellet-supplemented groups. A significant increase in mean body weight of 14% in the HFD group is considered obese (17).

### 2.9 Mating

Upon confirmation of obesity in HFD groups, all rats in both normal chow-fed rats and HFD-fed groups were allowed to mate. This is done by placing female rats with fertile male rats in the ratio of 1:1 until successful copulation. Each day, vaginal smear was performed in female rats at approximately 8–10 a.m. to confirm pregnancy, and the smears were observed under a microscope with a ×100 magnification (KF2; Carl Zeiss, Hamburg, Germany). Detection of sperm indicates successful mating. The first sperm detection has been recorded as 0 post-coitum (18).

### 2.10 Gestation and weaning

The treatment with *E. tapos* yoghurt initiated from the first day of gestation until postnatal day (PND) 21. The obese dam’s treatment groups are as follows: normal chow and saline (NS), HFD and saline (HS), HFD and yoghurt (HY), HFD and 5 mg/kg of *E. tapos* yoghurt (HYT5), HFD and 50 mg/kg of *E. tapos* yoghurt (HYT50), and HFD and 500 mg/kg of *E. tapos* yoghurt (HYT500).

### 2.11 Body weight and calorie intake

Changes in body weight were documented weekly for all dams and 24-h food intake (kJ) and calorie intake were measured weekly according to the method describe elsewhere (17).

### 2.12 Plasma lipid profile, renal profile, liver profile, and leptin analysis

At the end of the study, on PND 21, all rats were fasted overnight prior to blood taking. For the blood-taking procedure, 5 ml of blood was collected in heparin tubes via cardiac puncture. The tubes were then centrifuged at 3,500 rpm for 15 min to obtain the plasma. Plasma was collected into a plain tube and stored at −20°C. Plasma lipid profiles [total cholesterol (TC), triglycerides (TG), low-density lipoprotein (LDH), and high-density lipoprotein (HDL)] were analyzed with a diagnostic reagent test kit (Roche, Germany) using Hitachi Automatic Analyzer 902 (Tokyo, Japan) (19). The serum leptin levels were measured based on the rat leptin ELISA kit provided by MyBioSource. The assays were performed according to the guideline provided with the sensitivity maintained at 1.0 ng/ml (20). Plasma was further analyzed for renal and liver profile using Alere Cholestech LDX® Analyzer (Alere, UK).

### 2.13 Histopathological analysis

Organs such as liver, kidney, colon, and fat tissue (visceral, retroperitoneal white adipose tissue) were collected after euthanizing all rats with carbon dioxide overdose. Tissue processing and sectioning were done on all preserved organs until a thin layer of paraffin ribbon is obtained, which was then placed in the water bath before being transferred to glass slides. All slides were then stained with hematoxylin and eosin (H&E) stain prior to histological viewing under the microscope (21). The presence of lesions and any form of abnormalities has been verified by a certified histopathologist from Universiti Putra Malaysia.

### 2.14 Statistical analysis

Statistical analyses were performed using SPSS 27.0, and the results were expressed as mean ± SEM. All data were tested for normality and compared using one-way analysis of variance followed by *post-hoc* least significant difference. *p*-value < 0.05 was considered significant. Comparison data between genders were analyzed by Student’s independent *t*-test while Pearson correlation test was used to find the correlation between body weight and calorie intake.

## 3 Results

### 3.1 Analysis of bioactive compounds of *E. tapos* yoghurt

**Figure 1:**
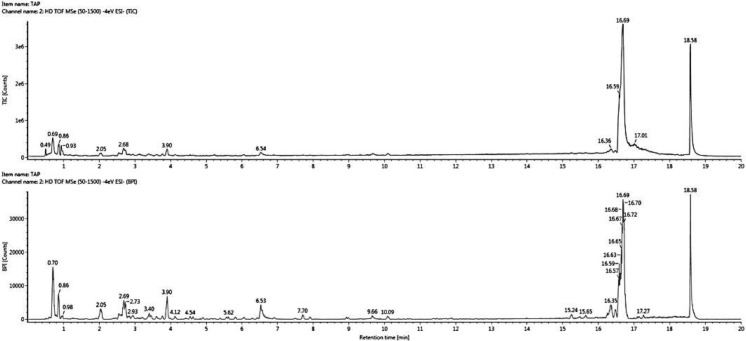
Comprehensive peak characterization trace showing bioactive compounds present in *E. tapos* yoghurt from TWIMS-QTOFMS analysis.

**Figure 1** shows the chromatograms of bioactive compound quantification from 1 ml of *E. tapos* yoghurt and its peak maxima. The peak maxima were observed at 16.69 s followed by 18.58 s. **Table 1** shows the isolated bioactive compounds from *E. tapos* yoghurt. All identified bioactive compounds from *E. tapos* yoghurt have been classified according to the class they belong to in **Table 1**. There are more than 12 classes of bioactive compounds identified from *E. tapos* yoghurt. The classes identified include polyphenols, glycosides, glucosides, alkaloids, flavonoids, phenolic acids, carbohydrates, amino acids, isoprenes, organic compounds, piscidic acids, and dicarboxylic acids. According to the TWIMS-QTOFMS analysis, *E. tapos* yoghurt as in **Figure 1** shows various peaks at different time intervals. The highest peak was recorded at a retention time of 16.69 min for isomaltose, a disaccharide.

**Table 1:** Bioactive compounds of *E. tapos* yoghurt.

Compound	Formula	Class	Molecular weight (Da)	Observed molecular weight (Da)	Observed m/z	Mass error (ppm)	Observed RT (min)
Polyphenol							
1,2,3,4,6-Penta-O-galloyl-β-D-glucopyranoside	C_41_H_32_O_26_	Gallotannin	940.11818	940.1212	939.114	3.2	9.67
1,2,3,6-Tetra-O-galloyl-β-D-glucopyranoside	C_34_H_28_O_22_	Tannins	788.10722	788.1098	787.1025	3.2	8.57
E-p-Coumatic acid	C_9_H_8_O_3_	Dietary polyphenol	164.04734	164.0471	163.0398	−1.5	1.07
Glycosides							
Scropolioside A	C_35_H_44_O_18_	Monoterpenoids Iridoid glycosides	752.25276	752.251	751.2437	−2.4	8.84
Yadanzioside A	C_32_H_44_O_16_	Quassinoid glycoside	684.26294	684.2649	683.2576	2.9	7.72
Forsythoside D	C_20_H_30_O_13_	Phenylethanoid glycosides	478.16864	478.1685	477.1612	−0.3	6.02
Rehmannioside A	C_21_H_32_O_15_	Carotenoid glycoside	524.17412	524.174	523.1668	−0.2	6.02
2,3,5,4’-Tetrahydroxystilbene-2-O-(6’’-O-acetyl)-β-D-glucopyranoside	C_22_H_24_O_10_	Glycoside	448.13695	448.1364	447.1292	−1.2	2.73
(Z)-(1S,5R)-β-Pinen-10-yl-β-vicianoside	C_21_H_34_O_10_	Monoterpene glycoside	446.2152	446.2155	445.2082	0.6	6.25
4-Methoxybenzal-dehyde-2-O-β-D-xylosyl(1→6)β-D-glucopyranoside	C_19_H_26_O_12_	Glycoside	446.14243	446.1425	445.1352	0.2	3.76
Asperulosidic acid	C_18_H_24_O_12_	Glycoside Iridoid monoterpenoid	432.12678	432.1269	431.1196	0.3	4.2
Neolinustatin	C_17_H_29_NO_11_	Cyanogenic glycosides	423.17406	423.1743	422.167	0.5	3.9
Fuzinoside	C_15_H_28_O_13_	Disaccharide glycoside	416.15299	416.1531	415.1459	0.3	1.88
Asperuloside	C_18_H_22_O_11_	Iridoid monoterpenoid glycoside	414.11621	414.1166	413.1093	1	10.12
Bruceine E_1	C_20_H_28_O_9_	Quassinoid	412.17333	412.1732	411.166	−0.3	15.95
Linustatin	C_16_H_27_NO_11_	Cyanogenic glycoside	409.15841	409.1585	408.1513	0.3	2.94
Erythro-dihydroxyde-hydrodiconiferyl alcohol	C_20_H_24_O_8_	Unidentified	392.14712	392.1461	391.1389	−2.5	4.31
Apocynoside I	C_19_H_30_O_8_	Ionone glucoside	386.19407	386.1941	385.1868	0	6.74
Calycanthoside	C_17_H_20_O_10_	Isofraxidin-7-glucoside	384.10565	384.1057	383.0984	0.1	8.06
1-O-Caffeoyl-β-D-glucopyranoside	C_15_H_18_O_9_	Cinnamate ester Hydroxycinnamic acid glycosides	342.09508	342.0956	341.0884	1.6	5.29
Glucoside							
Loroglossin	C_34_H_46_O_18_	Phenolic glucoside	742.26841	742.27	741.2628	2.2	9.33
Picrasinoside B	C_28_H_40_O_11_	Quassinoid glucoside					
Picrasinoside H	C_30_H_44_O_13_	Quassinoid glucoside	552.25706	552.2543	551.2471	−5	15
Loganic acid-6’-O-β-D-glucoside	C_22_H_34_O_15_	Iridoidal glucoside	612.27819	612.2787	611.2714	0.8	13.52
19β-Glucosyl-14-deoxyandrographoside	C_26_H_40_O_9_	Diterpene glucoside	496.26723	496.2665	495.2592	−1.5	6.85
Sinapaldehyde glucoside	C_17_H_22_O_9_	Glucoside	370.12638	370.1261	369.1189	−0.6	0.58
Alkaloid							
Ephedradine A	C_28_H_36_N_4_O_4_	Spermine alkaloid	492.27366	492.2738	491.2665	0.3	6.43
Ephedradine B	C_29_H_38_N_4_O_5_	Spermine alkaloid	522.28422	522.2844	521.2771	0.3	9.29
Ephedradine C	C_30_H_40_N_4_O_5_	Spermine alkaloid	536.29987	536.3006	535.2933	1.3	9.53
Flazin	C_17_H_12_N_2_O_4_	Harmala alkaloid	308.07971	308.0793	307.072	−1.4	12.84
Fawcettiine	C_18_H_29_NO_3_	Lycopodine-type alkaloid	307.21474	307.2142	306.207	−1.6	13.22
Tribulusterine	C_16_H_12_N_2_O_2_	β-Carboline alkaloid	264.08988	264.0894	263.0821	−2	12.84
Guanine	C_5_H_5_N_5O_	Purine bases	151.04941	151.0489	150.0416	−3.4	1.92
Phenolic acid							
1,3,5-O-Tricaffeoyl-quinic acid	C_34_H_30_O_15_	Quinic acids	678.15847	678.1552	677.148	−4.8	0.87
Ligustrosidic acid	C_26_H_32_O_13_	Phenols–Monophenols Terpenoids–Iridoids	552.18429	552.1851	551.1778	1.5	7.07
Flavonoids							
Kaempferol 3-O-α-L-rhamnopyranosyl-(1→2)-β-D-glucuronopyranoside	C_27_H_28_O_16_	Kaempferol flavonol	608.13773	608.1352	607.1279	−4.1	0.85
5′-Methoxy-bilobetin	C_32_H_22_O_11_	Bioflavonoids Polyflavonoids	582.11621	582.1163	581.109	0.2	0.97
Acacetin-7-O-(6”-O-acetyl)-β-D-glucopyranoside	C_24_H_24_O_11_	Flavonoids	488.13186	488.1311	487.1239	−1.5	0.66
2-Methoxy-4-acetylphenol 1-O-α-L-rhamnopyranosyl-(1’’→6’)-β-D-glucopyranoside	C_21_H_30_O_12_	Flavone	474.17373	474.1744	473.1672	1.5	5.83
Astragaline E	C_14_H_16_N_2_O_5_	Kaempferol-3-O-β-d-glucoside Flavonoid	292.10592	292.1059	291.0986	−0.2	4.55
2′-Hydroxy-4′,6′-dimethoxydihydrochalcone	C_17_H_18_O_4_	Dihydrochalcones 2’-hydroxychalcones Flavonoid	286.12051	286.1204	285.1131	−0.4	13.97
Carbohydrate							
Mannotriose	C_18_H_32_O_16_	Oligosaccharides	504.16903	504.1696	503.1624	1.2	0.66
Isomaltose	C_12_H_22_O_11_	Disaccharide	342.11621	342.1161	341.1089	−0.2	0.69
Pentose	C_5_H_10_O_5_	Monosaccharide	150.05282	150.0524	149.0451	−2.9	0.7
Galactose	C_6_H_12_O_6_	Monosaccharide sugar	180.06339	180.0629	179.0556	−2.9	0.7
Meso-inositol	C_6_H_12_O_6_	Carbocyclic sugar Monosaccharide	180.06339	180.0627	179.0555	−3.6	2.55
α-D-(6-O-4-Methyl-3,5-dimethoxycinnamoyl)-glucopyranosyl(1→2)-β-D-(3-O-sinapoyl)-fructofuranose	C_35_H_44_O_18_	Sugar	752.25276	752.2515	751.2442	−1.7	8.24
Amino acids							
Tyrosine	C_9_H_11_NO_3_	Nonessential amino acid	181.07389	181.0735	180.0662	−2.1	1.07
Indigoticoside A	C_26_H_34_O_11_	Phenylpropanoid	522.21011	522.2104	521.2031	0.5	9.79
Segetalin B	C_24_H_32_N_6_O_5_	Cyclopentapeptide	484.24342	484.244	483.2368	1.3	14.53
Isoprene							
Bruceine B	C_23_H_28_O_11_	Triterpenoid	480.16316	480.165	479.1578	3.9	15.25
9,16-Dioxyhydroxy-10,12,14-triene-18 carbonic acid	C_18_H_30_O_4_	Terpene	310.21441	310.2137	309.2064	−2.3	14.89
Organic compound							
Sanleng acid	C_18_H_34_O_5_	Organic acid compounds	330.24062	330.2391	329.2319	−4.5	15.44
Methylsuccinic acid	C_5_H_8_O_4_	Organic compound	132.04226	132.0418	131.0345	−3.7	0.7
Medicagol	C_16_H_8_O_6_	Coumestans	296.03209	296.0323	295.025	0.6	0.96
Sinapic acid	C_11_H_12_O_5_	Hydroxycinnamic acid Phenylpropanoid	224.06847	224.0679	223.0606	−2.7	5.92
Arteamisinine I	C_13_H_18_O_2_	Sesquiterpene lactone	206.13068	206.1302	205.1229	−2.3	5.23
Mono-ethyl fumarate	C_6_H_8_O_4_	Fumaric acid monoethyl ester	144.04226	144.0417	143.0344	−3.8	0.7
5R-5-Hydroxymethyl-2(5H)-furanone	C_5_H_6_O_3_	–	114.03169	114.0312	113.0239	−4.2	0.7
Piscidic acid							
Piscidic aciddiethyl ester	C_15_H_20_O_7_	Piscidic acid	312.1209	312.1209	311.1136	0	5.23
Dicarboxylic acids							
Sebacic acid	C_10_H_18_O_4_	Dicarboxylic acid	202.12051	202.1198	201.1125	−3.4	12.39
Phenylpropionic acid	C_9_H_11_NO_2_	Carboxylic acid	165.07898	165.0785	164.0712	−2.9	2.54
Eucommiol	C_9_H_16_O_4_	Carboxylic acid	188.10486	188.1045	187.0972	−2.1	9.72
Uncategorized							
3’,4’,7-Tribenzylepisappanol	C_37_H_34_O_6_		574.23554	574.2374	573.2302	3.3	2.54

### 3.2 Changes in body weight and total calorie intake in dams after obesity induction prior to mating

**Figure 2:**
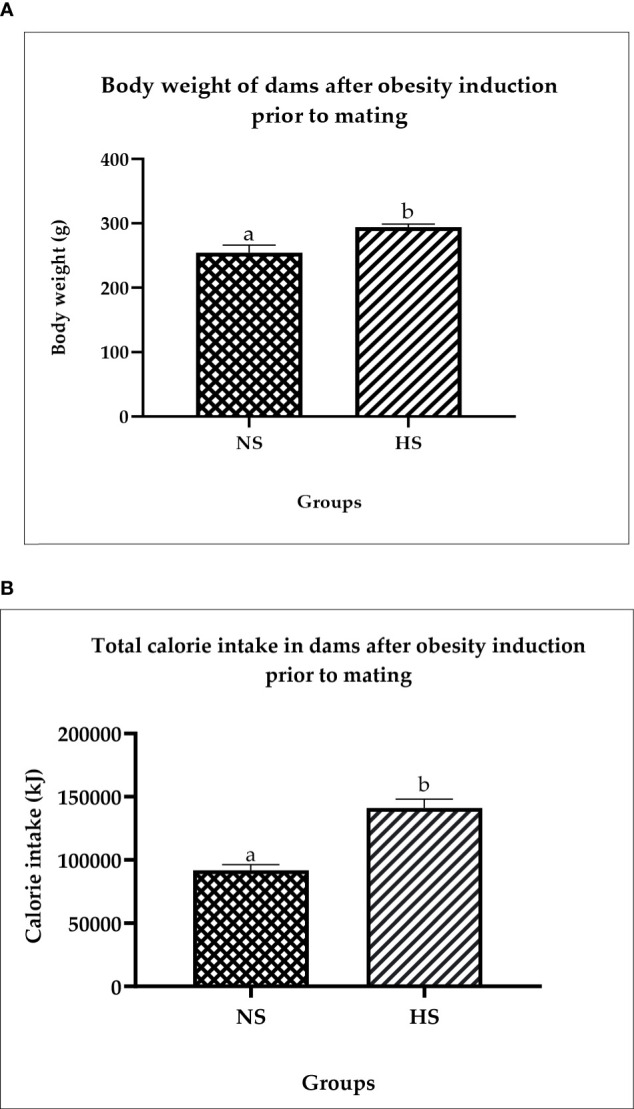
**(A)** Changes in body weight in dams after obesity induction prior to mating. NS: normal chow and saline; HS: HFD and saline. Values are expressed as mean ± SEM. Different letters indicate a significant difference at *p* < 0.05. **(B)** Total calorie in dams after obesity induction prior to mating. NS, normal chow and saline; HS, HFD and saline. Values are expressed as mean ± SEM. Different letters indicate a significant difference at *p* < 0.05.

**Figure 2A** shows the changes in body weight before and after obesity induction. The HS groups show significantly (p < 0.05) higher body weight (14%) compared to NS. **Figure 2B** shows the total calorie intake after obesity induction prior to mating. The result shows that the calorie intake in HS groups is significantly high (*p* < 0.05) compared to the NS group. The Pearson correlation shows a strong positive correlation (r = 0.92) between calorie intake and body weight.

### 3.3 Changes in body weight and total calorie intake in dams during weaning

**Figure 3:**
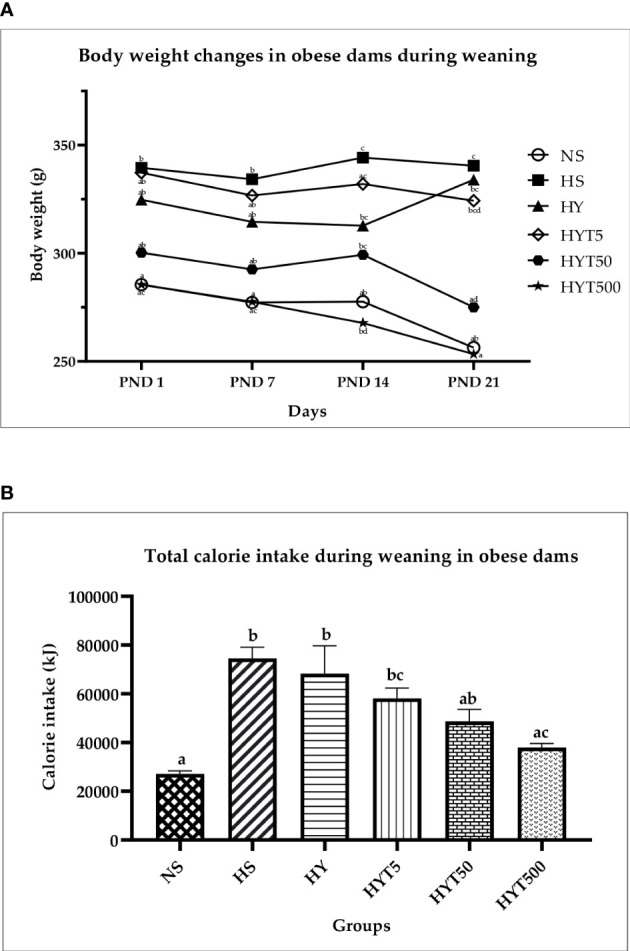
**(A)** Changes in body weight of dams during weaning. NS: normal chow and saline; HS: HFD and saline; HY: HFD and yoghurt; HYT5: HFD and 5 mg/kg of *E tapos* yoghurt; HYT50: HFD and 50 mg/kg of E tapos yoghurt; HYT500: HFD and 500 mg/kg of *E tapos* yoghurt. Values are expressed as mean ± SEM. Different letters indicate a significant difference at *p* < 0.05. **(B)** Total calorie intake in obese dams during weaning. NS, normal chow and saline; HS, HFD and saline; HY, HFD and yoghurt; HYT5, HFD and 5 mg/kg of *E tapos* yoghurt; HYT50, HFD and 50 mg/kg of *E tapos* yoghurt; HYT500, HFD and 500 mg/kg of *E tapos* yoghurt. Values are expressed as mean ± SEM. Different letters indicate a significant difference at *p* < 0.05.

**Figure 3A** shows the changes in obese dam’s body weight during weaning. The results show that the body weight of HS on PND 1, 7, 14, and 21 is significantly higher (p < 0.05) compared to NS. However, on PND 1, 7, 14, and 21, the HYT500-treated group shows significant (*p* < 0.05) reduction in body weight compared to HS. The mean value of HYT500 is similar to NS. There is no significant (p > 0.05) difference between the HY-, HY5-, and HYT50-treated groups on PND 1, 7, and 14. However, on PND 21, the body weight in HYT50 shows significant (p < 0.05) reduction compared to the HS group and the mean value of HYT50 is similar to NS. **Figure 3B** shows the total calorie intake during weaning. The data show that there is a significant increase (p < 0.05) of calorie intake in HS, HY, HYT5, and HYT50 groups compared to the NS, while there is a significant decrease (p < 0.05) of calorie intake in the HYT500 group compared to HS. There is a significant decrease (*p* < 0.05) of calorie intake in the HYT500 group compared to HY. The mean value for total calorie intake in HYT500 is similar to the NS group. Pearson correlation shows a strong positive correlation (*r* = 0.89) between total calorie intake and body weight in obese dams during weaning up to PND 21.

### 3.4 The effect of *E. tapos* yoghurt on plasma leptin of obese dams on PND 21

**Figure 4:**
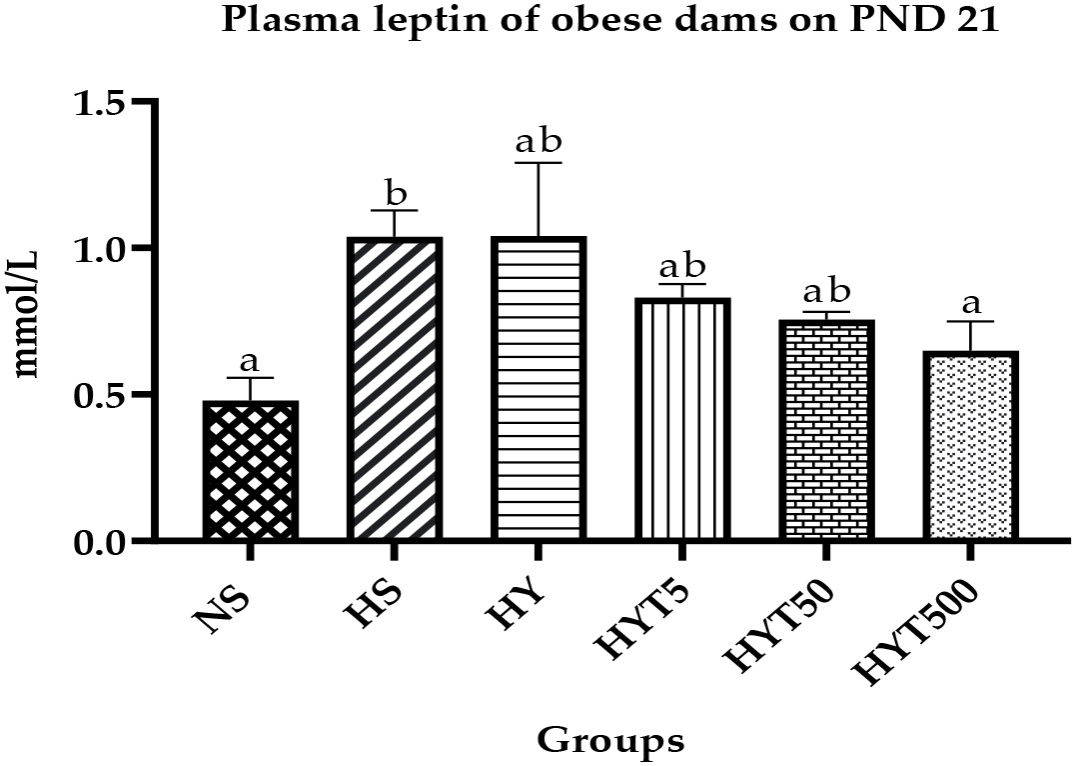
Plasma leptin level in obese dams on PND 21. NS, normal chow and saline; HS, HFD and saline; HY, HFD and yoghurt; HYT5, HFD and 5 mg/kg of *E. tapos* yoghurt; HYT50, HFD and 50 mg/kg of *E. tapos* yoghurt; HYT500, HFD and 500 mg/kg of *E. tapos* yoghurt. Values are expressed as mean ± SEM. Different letters indicate a significant difference at *p* < 0.05.

**Figure 4** shows the plasma leptin level of obese dams. The data show that the plasma leptin level of HS is significantly high (*p* < 0.05) compared to the NS group. There is no significant (*p* > 0.05) difference among HY, HYT5, and HYT50 groups on plasma leptin level. However, there is a significant (*p* < 0.05) reduction in plasma leptin level of the highest *E. tapos* yoghurt supplemented group (HYT500), and the mean value of HYT500 is comparable to that of the NS group on PND 21.

### 3.5 The effect of *E. tapos* yoghurt on obese dam’s renal profile on PND 21

**Table 2** shows the effect of *E. tapos* yoghurt on the obese dam’s renal profile. The data show that the plasma level of urea and creatinine is significantly high (p < 0.05) in the HS group compared to the NS group. However, the treatment with *E. tapos* yoghurt with all three different concentrations restores the plasma level of urea and creatinine in obese dams. In this setting, the HYT5, HYT50, and HYT500 groups show a significantly low (*p* < 0.05) level of urea compared to the HS group. The mean value of HYT5, HYT50, and HYT500 for plasma urea level correlates with the NS group’s mean value. Meanwhile, the HYT50 and HYT500 groups show a significantly low (p < 0.05) level of creatinine in plasma compared to the HS group. The mean value for plasma creatinine level for the HYT50 and HYT500 groups was almost similar to that of the NS group. However, there is no significant (p > 0.05) difference between NS, HS, HY, HYT5, and HYT50 on sodium (Na), potassium (K), and chloride (Cl^−^) levels in plasma of obese dams.

**Table 2:** Renal profile obese dams after treated with *E. tapos* yoghurt.

	Na (mmol/L)	K (mmol/L)	Cl^−^ (mmol/L)	Urea (mmol/L)	Creatinine (mmol/L)
NS	143.50 ± 1.25	9.30 ± 0.67	99.00 ± 1.08	7.16 ± 0.37 ^a^	54.40 ± 2.77 ^a^
HS	144.00 ± 0.71	8.54 ± 0.71	100.20 ± 0.97	9.63 ± 0.61 ^b^	65.00 ± 2.30 ^b^
HY	145.20 ± 0.58	7.74 ± 0.52	99.60 ± 1.44	6.86 ± 0.68 ^a^	61.75 ± 2.06 ^ab^
HYT5	143.40 ± 0.68	7.54 ± 0.37	99.80 ± 0.80	7.54 ± 0.83 ^a^	57.20 ± 3.46 ^ab^
HYT50	143.40 ± 1.36	8.20 ± 0.45	102.60 ± 1.12	6.48 ± 0.63 ^a^	53.40 ± 2.91 ^a^
HYT500	143.20 ± 0.49	7.92 ± 0.45	102.60 ± 1.86	6.16 ± 0.64 ^a^	54.80 ± 2.71 ^a^

NS, normal chow and saline; HS, HFD and saline; HY, HFD and yoghurt; HYT5, HFD and 5 mg/kg of *E. tapos* yoghurt; HYT50, HFD and 50 mg/kg of *E. tapos* yoghurt; HYT500, HFD and 500 mg/kg of *E. tapos* yoghurt; Na, sodium; K, potassium; Cl^−^, chloride. Values are expressed as mean ± SEM. Different letters indicates a significant difference at p < 0.05.

### 3.6 The effect of *E. tapos* yoghurt on obese dam’s liver enzymes on PND 21

**Table 3** shows the effect of *E. tapos* yoghurt on obese dam’s liver enzymes on different groups. The data show no significant difference (p > 0.05) on total protein between NS, HS, HY, HYT5, HYT50, and HYT500 groups. The level of albumin and total bilirubin is significantly low (p < 0.05) in the HS group compared to the NS group. However, the high *E. tapos* concentration (HYT500) shows a significant increase in albumin level while the HYT50 and HYT500 groups show a significant increase (p < 0.05) in total bilirubin compared to the HS group. The mean value of HYT500 for albumin level and that of HYT50 and HYT500 for total bilirubin level are almost similar to the NS group. There is no significant (p > 0.05) difference among HY, HYT5, and HYT50 compared to the HS group for plasma albumin level. Meanwhile, the levels of globulin, albumin/globulin ratio, alkaline phosphatase (ALP), aspartate aminotransferase (AST), alanine transaminase (ALT), and gamma-glutamyl transferase are significantly (p < 0.05) high in the HS group compared to the NS group. For globulin, the high concentration of *E. tapos* yoghurt (HYT500) significantly reduced the level of plasma globulin with an identical mean value to the NS group. There is no significant difference among HYT5 and HYT50 compared to the HS group for plasma globulin level. The HY group shows a significantly (p < 0.05) high level of globulin compared to the NS group and no significant (p > 0.05) difference compared to the HS group. For the albumin/globulin ratio, the mean value of HY and HYT5 is significantly (p < 0.05) high compared to the HS group. However, HYT50 and HYT500 show gradual reduction in plasma albumin/globulin ratio, yet there is no significant (p > 0.05) difference among HYT50 and HYT500 compared to the HS and NS groups. The level of plasma ALP is significantly (p < 0.05) low in the HY group compared to the HS group. However, the ALP level remains significantly (p < 0.05) high compared to the NS group. All three different concentrations of *E. tapos* yoghurt (HYT5, HYT50, and HYT500) significantly (p < 0.05) decrease the plasma ALP level compared to the HS group with a mean value similar to the NS group. The plasma level of AST is significantly (p < 0.05) low in the HY, HYT5, HYT50, and HYT500 groups compared to the HS group, with an identical mean value to the NS group. There is no significant (p > 0.05) difference among HY, HYT5, HYT50, and HYT500 in plasma AST level. Meanwhile, the HY group shows a significantly (p < 0.05) high level of ALT compared to the NS group. However, all three different concentrations of *E. tapos* yoghurt (HYT5, HYT50, and HYT500) significantly (p < 0.05) decrease the plasma ALT level compared to the HY group. There is no significant difference (p > 0.05) between HYT5, HYT50, and HYT500 compared to the HS or NS group. The level of gamma-glutamyl transferase is significantly (p < 0.05) decreased in HY, HYT5, and HYT500 compared to the HS group, with a mean value almost similar to the NS group. However, there is no significant difference (p > 0.05) recorded in the HYT50 group in comparison with the HY or NS group. The data for total bilirubin were significantly (p < 0.05) low for both HY and HYT5 compared to the HS group. However, HYT50 and HYT500 show a significant (p < 0.05) increase in total bilirubin level of plasma compared to the HS group. The mean value of HYT50 and HYT500 is similar to the NS group.

**Table 3:** Liver enzyme level in obese dams after treated with *E. tapos* yoghurt.

	Total protein (g/L)	Albumin (g/L)	Globulin (g/L)	Albumin–globulin ratio	ALP (U/L)	AST (U/L)	ALT (U/L)	Gamma-Glutamyl Transferase (U/L)	Total Bilirubin (μmol/L)
NS	78.25 ± 2.78	42.00 ± 0.58 ^a^	33.33 ± 0.33 ^a^	0.92 ± 0.01 ^a^	131.67 ± 0.67 ^ab^	139.67 ± 1.33 ^a^	65.00 ± 0.58 ^a^	1.33 ± 0.33 ^a^	2.33 ± 0.33 ^a^
HS	75.40 ± 1.12	36.67 ± 1.20 ^b^	37.67 ± 0.33 ^b^	1.17 ± 0.05 ^b^	210.00 ± 14.00 ^c^	240.67 ± 4.91 ^b^	88.33 ± 5.92 ^bc^	6.67 ± 0.33 ^b^	1.00 ± 0.00 ^b^
HY	77.00 ± 2.53	38.33 ± 0.67 ^ab^	38.67 ± 3.38 ^b^	1.17 ± 0.03 ^b^	187.33 ± 20.73 ^c^	143.00 ± 14.74 ^a^	104.67 ± 7.54 ^c^	3.00 ± 1.53 ^a^	1.00 ± 0.00 ^b^
HYT5	79.40 ± 1.03	40.33 ± 1.20 ^ab^	35.67 ± 0.33 ^ab^	1.13 ± 0.12 ^b^	139.33 ± 17.84 ^bd^	124.67 ± 10.20 ^a^	80.00 ± 7.02 ^ab^	3.00 ± 1.53 ^a^	1.00 ± 0.00 ^b^
HYT50	74.20 ± 0.97	39.67 ± 0.67 ^ab^	34.33 ± 0.33 ^ab^	1.06 ± 0.02 ^ab^	97.67 ± 4.81 ^a^	135.00 ± 4.16 ^a^	74.00 ± 7.51 ^ab^	4.33 ± 1.67 ^ab^	2.33 ± 0.33 ^a^
HYT500	74.80 ± 3.07	42.33 ± 0.88 ^a^	32.00 ± 0.58 ^a^	1.06 ± 0.03 ^ab^	124.33 ± 13.86 ^ab^	132.67 ± 11.05 ^a^	76.33 ± 7.54 ^ab^	1.33 ± 0.33 ^a^	2.67 ± 0.33 ^a^

NS, normal chow and saline; HS, HFD and saline; HY, HFD and yoghurt; HYT5, HFD and 5 mg/kg of *E. tapos* yoghurt; HYT50, HFD and 50 mg/kg of *E. tapos* yoghurt; HYT500, HFD and 500 mg/kg of *E. tapos* yoghurt; ALP, alkaline phosphatase; AST, aspartate aminotransferase; ALT, alanine transaminase. Values are expressed as mean ± SEM. Different letters indicate a significant difference at p < 0.05.

### 3.7 The effect of *E. tapos* yoghurt on obese dam’s lipid profile on PND 21

**Table 4** shows the effect of *E. tapos* yoghurt on the obese dam’s lipid profile. The data show that the level of HDL is significantly low (p < 0.05) while the levels of non-HDL, LDL, and triglyceride are significantly high (p < 0.05) in the HS group compared to the NS group. However, the treatment with *E. tapos* yoghurt with all three different concentrations restores the lipid profile level in obese dams. The HYT500 group shows a significantly high level of HDL and low level of LDL, non-HDL, and triglyceride compared to the HS group. The mean value of the HYT500 group is almost similar to the NS group. However, there is no significant (p > 0.05) difference between HS, HY, HYT5, and HYT50 in lipid analyses.

**Table 4:** Lipid profile obese dams after treated with *E. tapos* yoghurt.

	Total cholesterol (mmol/L)	HDL (mmol/L)	Non-HDL (mmol/L)	LDL (mmol/L)	Triglyceride (mmol/L)
NS	1.70 ± 0.06 ^a^	0.73 ± 0.07 ^a^	0.53 ± 0.03 ^a^	0.70 ± 0.00 ^a^	0.75 ± 0.05 ^a^
HS	2.27 ± 0.08 ^b^	0.43 ± 0.03 ^b^	0.80 ± 0.00 ^b^	1.07 ± 0.03 ^b^	1.55 ± 0.25 ^b^
HY	1.93 ± 0.03 ^ab^	0.57 ± 0.08 ^ab^	0.67 ± 0.03 ^ab^	0.97 ± 0.07 ^ab^	1.00 ± 1.00 ^ab^
HYT5	2.00 ± 0.06 ^ab^	0.57 ± 0.03 ^ab^	0.67 ± 0.03 ^ab^	0.90 ± 0.10 ^ab^	0.90 ± 1.00 ^ab^
HYT50	2.07 ± 0.14 ^ab^	0.63 ± 0.08 ^ab^	0.63 ± 0.15 ^ab^	0.87 ± 0.09 ^ab^	0.75 ± 0.05 ^a^
HYT500	1.60 ± 0.15 ^a^	0.73 ± 0.03 ^a^	0.60 ± 0.00 ^a^	0.70 ± 0.10 ^a^	0.80 ± 0.10 ^a^

NS, normal chow and saline; HS, HFD and saline; HY, HFD and yoghurt; HYT5, HFD and 5 mg/kg of *E. tapos* yoghurt; HYT50, HFD and 50 mg/kg of *E. tapos* yoghurt; HYT500, HFD and 500 mg/kg of *E. tapos* yoghurt; HDL, high-density lipoprotein; LDL, low-density lipoprotein. Values are expressed as mean ± SEM. Different letters indicate a significant difference at p < 0.05.

### 3.8 The effect of *E. tapos* yoghurt on obese dam’s organs on PND 21

**Table 5** shows the effect of *E. tapos* yoghurt on obese dam’s organs up to PND 21. The data show a significant increase (p < 0.05) in the liver, kidney, retroperitoneal white adipose tissue (RpWAT), visceral weight, and the length of colon in the HS group compared to the NS group. *E. tapos* yoghurt treatment of HYT50 and HYT500 shows a significant decrease (p < 0.05) in liver weight compared to HS group. The mean value of HYT50 and HYT500 for liver weight is almost similar to the NS group. However, liver weight in the HY and HYT5 groups remains significantly high compared to the NS group with a mean value similar to the HS group. The kidney weight shows a significant decrease (p < 0.05) in the HYT5, HYT50, and HYT500 groups compared to the HS group. There is no significant difference (p > 0.05) in the HYT5, HYT50, and HYT500 groups compared to the NS group for kidney weight. However, the HY group’s kidney is significantly higher (p < 0.05) than NS and shows no significant (p > 0.05) difference compared to the HS group. Colon weight shows no significant (p > 0.05) difference between the HS, HY, HYT5, HYT50, and HYT500 groups compared to the NS group. However, colon length is significantly (p < 0.05) higher in the HS and HY groups compared to the NS group. Meanwhile, colon length is significantly (p < 0.05) shorter in the HYT5, HYT50, and HYT500 groups compared to the HS and HY groups. There is no significant (p > 0.05) difference in colon length of HYT5, HYT50, and HYT500 compared to the NS group. Both retroperitoneal and visceral fat tissue are significantly (p < 0.05) heavier in the HS group compared to the NS group. There is a significant (p < 0.05) reduction in RpWAT and visceral fat in the HYT500 group compared to the HS group and the mean value of the HYT500 group is similar to that of the NS group. However, in HY5 and HY50, the weight of RpWAT and visceral remains significantly high (p < 0.05) compared to the NS group and there is no significant (p > 0.05) difference between HY5 and HY50 in RpWAT weight compared to the HS group.

**Table 5:** The changes in organ weight in obese dams after treated with *E. tapos* yoghurt.

	Liver (g)	Kidney (g)	Colon (g)	Colon length (cm)	RpWAT (g)	Visceral (g)
NS	8.33 ± 0.50 ^a^	1.66 ± 0.08 ^a^	1.97 ± 0.23	16.07 ± 1.85 ^a^	1.88 ± 0.37 ^a^	2.30 ± 0.23 ^a^
HS	11.02 ± 0.75 ^b^	2.24 ± 0.21 ^bc^	2.03 ± 0.12	26.67 ± 1.45 ^b^	4.41 ± 0.15 ^bc^	3.47 ± 0.18 ^bc^
HY	10.91 ± 0.30 ^b^	2.14 ± 0.12 ^cd^	2.58 ± 0.32	23.23 ± 0.79 ^b^	4.72 ± 0.57 ^c^	4.04 ± 0.37 ^c^
HYT5	10.95 ± 0.85 ^b^	1.80 ± 0.20 ^ad^	1.90 ± 0.12	18.83 ± 0.44 ^a^	3.39 ± 0.18 ^bd^	3.34 ± 0.54 ^bc^
HYT50	8.86 ± 0.57 ^a^	1.81 ± 0.05 ^ad^	1.87 ± 0.19	16.33 ± 1.33 ^a^	3.76 ± 0.62 ^bce^	3.06 ± 0.24 ^abc^
HYT500	8.06 ± 0.62 ^a^	1.73 ± 0.07 ^a^	2.06 ± 0.12	18.00 ± 2.08 ^a^	2.72 ± 0.16 ^ade^	2.66 ± 0.36 ^a^

NS, normal chow and saline; HS, HFD and saline; HY, HFD and yoghurt; HYT5, HFD and 5 mg/kg of *E. tapos* yoghurt; HYT50, HFD and 50 mg/kg of *E. tapos* yoghurt; HYT500, HFD and 500 mg/kg of *E. tapos* yoghurt; RpWAT, retroperitoneal white adipose tissue. Values are expressed as mean ± SEM. Different letters indicate a significant difference at p < 0.05.

### 3.9 The effect of *E. tapos* yoghurt on histological changes of obese dams on PND 21

**Figure 5:**
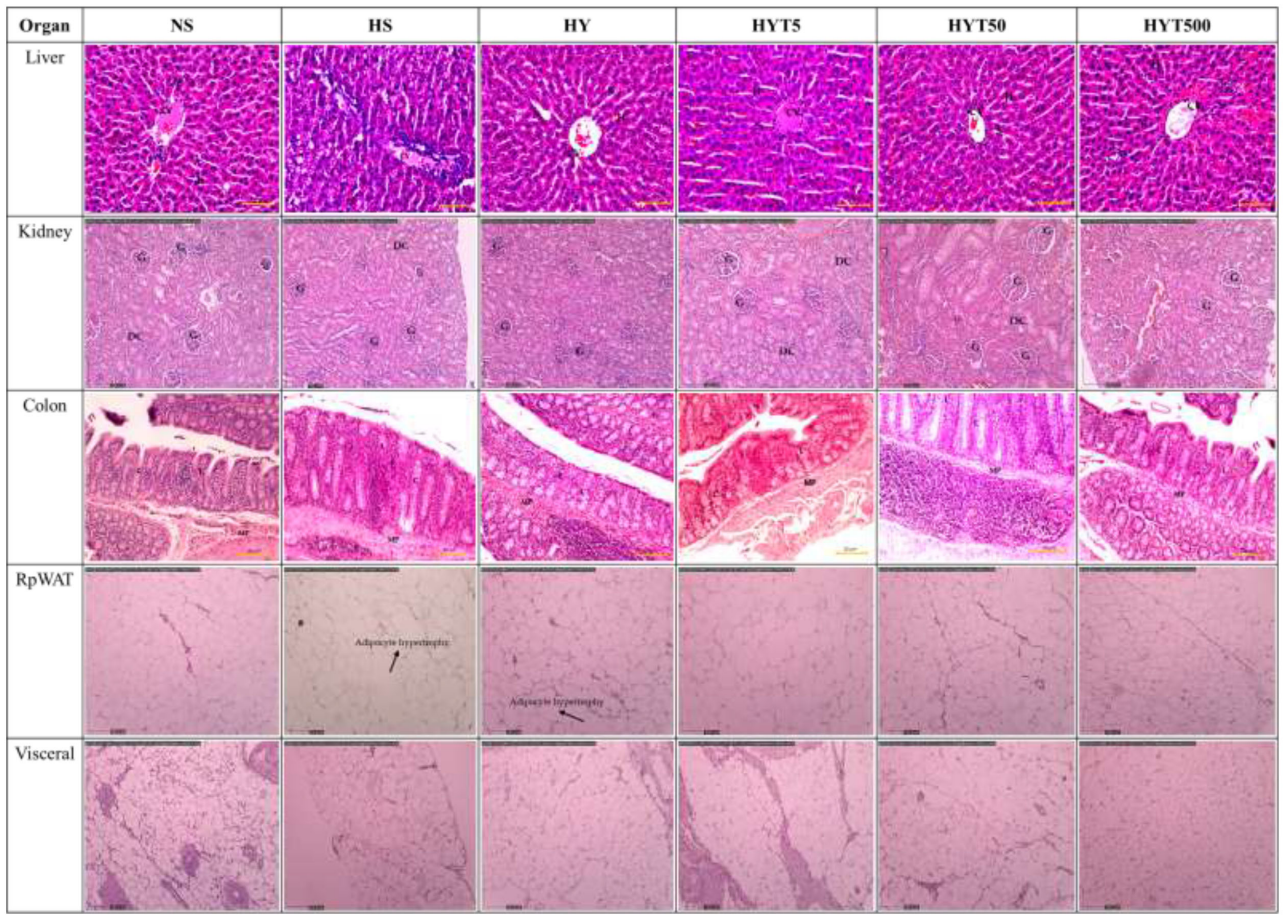
The effect of *E. tapos* yoghurt on the histological changes of kidney, liver, colon, RpWAT, and visceral of obese dams on PND 21. H, hepatocyte; CV, central vein; S, sinusoids; G, glomerulus; DC, distal convulated tubule; L, lamina propria; MP, mucosa; C, crypts of mucosa; RpWAT, retriperitoneal white adipose tissue.

**Figure 5** shows the effect of *E. tapos* yoghurt on the histological changes of kidney, liver, colon, RpWAT, and visceral of obese dams on PND 21. The histological analysis of kidney shows no abnormal lesion or tubular dilation. The glomerulus (G), distal convoluted tubule (DC), and renal corpuscle show normal appearance. Thus, no abnormalities were observed in kidney histology section in HS, HY, HYT5, HYT50, and HYT500 compared to the NS group. Meanwhile, the histological section of the liver in HS shows abnormal hepatocytes (H), sinusoids (s), and central vein (CV). The grading score for the HS group’s liver is recorded as 2 due to the presence of cell ballooning, more than 30% of cell steatosis, and lobular inflammation. The histological section of the HY group shows abnormal hepatocytes, sinusoids, and central vein. The grading score for the HY group liver is recorded as 1 due to the presence of cell ballooning and lobular inflammation and the absence of cell steatosis. However, the histological section of liver in NS, HYT5, HYT50, and HYT500 shows standard strands of hepatocytes, sinusoids, and central vein. It showed that 0% of biopsied hepatocytes were affected. The liver grading score for NS, HYT5, HYT50, and HYT500 was documented as 0 due to the absence of steatosis, lobular inflammation, and hepatocyte ballooning or lipid droplet. The histological section of the colon in HS shows severe detachment of epithelial cells, reduced mucosa content in the colonic wall, severe infiltration of lamina propria, severe inflammation, and the presence of fat deposition in the muscle layer, while submucosa and muscularis mucosa appear intact. The colonic tissue of the HY and HYT5 groups shows moderate level of infiltration in the lamina propria, moderate level of inflammation, and the presence of fat deposition in the muscle layer, while submucosa and muscularis mucosa appear intact. The colonic tissue of the HYT50 group shows a reduced level of infiltration in the lamina propria and inflammation compared to the HS group. Meanwhile, the colonic section of HYT500 shows a reduced level of infiltration in the lamina propria and fat deposition in the muscle layer compared to the HS group. The epithelial cell, mucosal content, and the architecture of submucosa and muscularis mucosa appear normal. The colonic tissue of the NS group shows normal architecture with normal appearance of epithelial cell, mucosal content, submucosa, and muscularis mucosa; the absence of infiltration in lamina propria; and no fat deposition in the muscle layer. The histological section of RpWAT and visceral of the HS group shows adipocyte expansion/hypertrophy of fat cells compared to NS. There are no abnormalities or hypertrophy of fat cells documented in RpWAT and visceral of HY, HYT5, HYT50, and HYT500 compared to NS. The histological structure of RpWAT and visceral of HY, HYT5, HYT50, and HYT500 appears similar to the NS group.

## 4 Discussion

Maternal obesity is the key predictor for many health conditions and the emergence of childhood obesity. Studies claim that maternal obesity is associated with the child’s adiposity level, and in turn might affect the neurodevelopmental progress. HFD consumption is one of the main reasons for obesity (22). Reportedly, a study proves that HFD intake increases the obese gene product known as leptin, total body fat, and calorie intake (23). This, in turn, manifests in the changes in histological and morphological changes of organs, liver, and renal markers (24). Thus, HFD is considered to be one of the most studied methods to induce obesity (22). In relevance to this claim, the difference of mean value of 14% in the HS group compared to the NS group proves the successful obesity induction in female SD rats prior to mating in the first phase of this study (17). In contrast, a recent study shows that *E. tapos* extract naturally possesses the anti-obesity effect in obese dams (25). However, the specific mechanism of how *E. tapos* yoghurt acts as an anti-obesity agent has not been discovered and yet to be revealed in this study. Hence, this study summarizes the specific effects of *E. tapos* yoghurt on total calorie intake, liver enzymes, renal enzymes, obese gene products, lipid profile, morphological and histological changes in liver, kidney, colon, and fat tissues.

In the second phase of this study, the HS group shows the highest level of calorie intake, body weight retention, and increased concentration of plasma leptin on PND 21. Studies shows that maternal obesity induces hypothalamic leptin resistance, and it is directly proportional to the calorie intake and body fat (26). In such a condition, satiety will be suppressed through the brain–gut axis (27), triggering the over-intake of nutrients, eventually leading to an increase in body weight, which often manifests as increased body weight or body mass index (28). Meanwhile, obese dams supplemented with the highest dosage of *E. tapos* concentration show gradual reduction in total calorie intake, body weight, and plasma leptin concentration on PND 21 in the HYT500 group. This could be due to the presence of 5′-methoxy-bilobetin, a bioflavonoid compound that is commonly used to treat hyperlipidemic condition. Likewise, bioflavonoid compounds have a vital role in suppressing lipid production, triglycerides, and total cholesterol level (29). Moreover, the presence of polyphenols in *E. tapos* further modulates adipohormones and the expression of gut peptide hormone, thereby suppressing the appetite (27), leading to decreased calorie intake and reduced level of body weight.

Furthermore, obese dams without intervention in this study show a high level of urea and creatinine in the plasma while *E. tapos* yoghurt-supplemented obese dams have shown reduction in plasma urea and creatinine levels. These results are similar to those of the previous study that proves that the HFD-supplemented group stimulates changes in renal function, by reducing glomerular filtration rate (GFR), thereby causing creatinine and urea level to rise in plasma (30). The main reason for this is the increased lipid level in HFD-supplemented dams, which, in turn, affects the renal function (31). In addition, HFD consumption could activate the lipogenic molecular pathways that may increase the level of triglycerides and the total cholesterol level as seen in the HS group’s lipid profile, causing lipid accumulation, which will lead to the reduced level of GFR in the end (32). On the flipside, the presence of high concentration of glycosides such as Asperuloside in *E. tapos* yoghurt is proven to regulate metabolic homeostasis by reversing HFD-induced obesity via the modulation of gut dysbiosis. A recent study further proves the effect of Asperuloside in treating HFD-induced obesity by improving metabolism in adipose tissue (33). Therefore, the high concentration of Asperuloside in *E. tapos* yoghurt could be one of the major reasons for the gradual reduction of triglycerides, non-HDL, total cholesterol, and LDL as shown in the lipid analysis of obese dams.

Besides, the increase of triglycerides, non-HDL, total cholesterol, and LDL in obese dams further influences the dysregulation of liver enzymes. As seen in the HS group, the level of albumin is significantly reduced while the level of globulin is increased compared to the NS group. This is because obesity is classified as a chronic inflammatory condition where the hypertrophy of adipocytes and hypoxia is a common clinical manifestation. In such condition, excessive level of tumor necrosis factor alpha (TNFα) will be produced, causing the albumin level to reduce (34) and the globulin level to increase (35). Henceforth, the imbalance of albumin/globulin ratio as seen in the HS group is one of the key predictors for the presence of inflammation due to fat tissue hypertrophy. Similarly, ALP is commonly expressed in adipocytes and ALP plays a vital role in the regulation of intracellular deposition of fats. As such, hypertrophy of fat tissue will stimulate the release of ALP into the blood circulation, causing the plasma ALP to rise in obese subjects (36). Comparably, obesity correlates positively with the plasma concentration of AST, ALT, and GGT (37), and inversely correlates with bilirubin (38). This could probably be due to the fact that obesity stimulates the release of excessive oxidative stress due to the presence of steatosis, ballooning, or inflammation in liver (38). As a result, DNA methylation in the liver will increase, indicating liver damage (39). In addition, excessive visceral fat secretes leptins and TNFα, which may further increase inflammation in the liver (40). Therefore, elevation of AST, ALT, and GGT is a common indicator in obesity (37) as seen in the HS group.

Conversely, treatment with *E. tapos* yoghurt (HYT500) in HFD-induced obesity dams propitiously increases the level of total serum bilirubin and albumin, and decreases the level of globulin, albumin–globulin ratio, ALT, AST, ALP, and GGT. The effectiveness of *E. tapos* yoghurt in regulating liver enzymes may be due to the presence of bioactive compounds that naturally possess anti-inflammatory properties such as Scropolioside A (41), Picrasinoside B (42), Ephedradine B (43), Indigoticoside A (44), Bruceine B (45), Asperulosidic acid (46), 2,3,5,4’-Tetrahydroxystilbene-2-O-(6’’-O-acetyl)-β-D-glucopyranoside (47), Astragaline E (48), and Sinapic acid (49). For instance, 2,3,5,4’-Tetrahydroxystilbene-2-O-(6’’-O-acetyl)-β-D-glucopyranoside could induce autophagy in liver via the activation of PI3K/Akt and Erk signaling pathway (50). In obesity, the supply of hypernutrition will deactivate AMPK, causing the activation of mTORC1, leading to the inactivation or suppression of autophagy. In such condition, lipid droplets will accumulate in the liver (51). As such, stimulation of autophagy will enhance lipid droplet removal and reduce the level of oxidative stress production as well as lipid peroxidation, thereby protecting the hepatocytes (52).

Furthermore, an increased level of triglyceride will cause fat deposition in the liver, leading to the development of abnormal hepatocytes, cell ballooning, inflammation, and scarring (53). This will eventually increase the weight of the liver as seen in the HS group’s morphological and histological analysis in this study. Lipid accumulation in the kidney may cause kidney weight to increase as well (30). In addition, studies show that the length of the intestine is longer in obese subjects compared to lean subjects (54). In fact, hypernutrition due to HFD intake compromises uncontrolled cell proliferation and length and depth of intestine villi, causing the length of the colon to increase (55). This is the main reason for the increase in colon length in the HS group in this study, which is further characterized by the detachment of epithelial cells and infiltration of fat in the submucosa. Hypertrophy of fat tissue in RpWAT and visceral in the HS group further defines the characteristics of obesity model in this study. In contrast, the oral administration of *E. tapos* yoghurt in HFD-induced maternal obese dams successfully reverses the abnormal condition in most of the organs. The presence of non-essential amino acids in *E. tapos* yoghurt such as tyrosine enhances lipid and carbohydrate metabolism, thereby promoting fat loss (56) in HFD-supplemented obese dams. As a result, HYT5-, HYT50-, and HYT500-supplemented groups’ organ histology appears to have an almost similar architecture to the NS group.

## 5 Conclusions

Maternal obesity is the root cause of childhood obesity. Thus, curbing maternal obesity is the best option to prevent the emerging critical cases of childhood obesity and its consequences. This study discovers the therapeutic effects of *E. tapos* yoghurt as an anti-obesity agent in maternal obese dams, specifically focusing into each possible parameters based on physiological, biochemical, and histological analysis. The bioactive compounds present in *E. tapos* yoghurt naturally possess the ability to suppress inflammation and appetite, thereby stimulating fat loss, and eventually causing gradual weight loss. From this study, we conclude that *E. tapos* yoghurt possesses no side effect. Hence, supplementation of *E. tapos* yoghurt during the gestational period up to weaning has been effective in the gradual weight loss of maternal obese dams from the 500-mg/kg-supplemented group in this study. As such, this study proves the effectiveness of the first medicinal plant-integrated yoghurt in curbing maternal obesity, and these data provide useful insights into considering natural probiotics such as yoghurt as a therapeutic agent to treat maternal obesity instead of modern drugs.

## Data availability statement

The original contributions presented in the study are included in the article/supplementary material. Further inquiries can be directed to the corresponding authors.

## Ethics statement

The animal study was reviewed and approved by All animal study was performed upon obtaining approval from Institutional Animal Care and Use Committee (IACUC), Universiti Putra Malaysia. The animal ethics committee granted approval for this study under the code UPM/IACUC/AUP-R025/2022.

## Author contributions

Conceptualization, RN and RR. Methodology, FO. Software, SB. Validation, HB, MY, and ST. Formal analysis, RN. Investigation, RN. Resources, AJ. Data curation, AA. Writing—original draft preparation, RN. Writing—review and editing, RN. Visualization, HE. Supervision, HB and MY. Project administration, SJ. Funding acquisition, HB and MY. All authors contributed to the article and approved the submitted version.
